# Myelin oligodendrocyte glycoprotein antibody-associated cerebral cortical encephalitis: a case report highlighting diagnostic challenges and therapeutic implications

**DOI:** 10.3389/fimmu.2025.1619807

**Published:** 2025-06-18

**Authors:** MengChan Liu, DaWei Li

**Affiliations:** Department of Neurology, The Fourth People’s Hospital of Shenzhen (Shenzhen Sami Medical Center), Shenzhen, China

**Keywords:** myelin oligodendrocyte glycoprotein-associated cerebral cortical encephalitis, seizures, headache, MRI, therapy, relapse

## Abstract

Myelin oligodendrocyte glycoprotein (MOG) antibody-associated disease (MOGAD) encompasses a spectrum of inflammatory demyelinating disorders of the central nervous system (CNS). Recognized clinical phenotypes include optic neuritis (ON), transverse myelitis (TM), acute disseminated encephalomyelitis (ADEM), brainstem encephalitis, aseptic meningitis, cortical encephalitis, demyelinating pseudotumor, and cranial nerve involvement. MOG antibody-associated cerebral cortical encephalitis (MOG-CCE) represents a rare but clinically significant subtype, often misdiagnosed due to heterogeneous clinical and neuroimaging features overlapping with other CNS disorders. We present a case of 36-year-old man with new-onset acute seizures and persistent headache. Initial brain magnetic resonance imaging (MRI) revealed no obvious signal abnormalities; however, subtle cortical swelling with sulcal effacement was identified in the left frontoparietal region, suggestive of focal cortical inflammation. Diagnostic workup revealed elevated MOG-IgG antibody titers in both serum and cerebrospinal fluid (CSF) using live cell-based assay. The patient demonstrated remarkable clinical response to combination immunotherapy with intravenous methylprednisolone and immunoglobulin. This case highlights the inclusion of MOG-CCE in the differential diagnosis of unexplained seizures and headaches accompanied by cortical swelling on neuroimaging, even in the absence of overt demyelinating lesions, and further underscores the critical role of early MOG-IgG testing and prompt immunotherapy to mitigate disease progression and improve neurological outcomes.

## Introduction

Myelin oligodendrocyte glycoprotein (MOG), a central nervous system (CNS)-specific transmembrane protein localized to the surface of oligodendrocye membranes, has gained recognition as a key autoantigen in neuroinflammatory disorders (1). Emerging evidence supports MOG antibody-associated disease (MOGAD) as a distinct clinical entity within the spectrum of autoimmune CNS conditions, characterized by IgG-mediated demyelination, stereotypic clinical-radiological phenotypes and differential therapeutic responses, compared to neuromyelitis optica spectrum disorder (NMOSD) (2, 3). Notably, the 2017 identification of cerebral cortical encephalitis associated with MOG-IgG seropositivity (MOG-CCE) expanded the phenotypic spectrum of MOGAD, revealing a distinct syndrome characterized by pharmacoresistant epilepsy accompanied by unilateral T2-weighted imaging (T2WI)-hyperintense cortical lesions on Magnetic Resonance Imaging (MRI) (4). Subsequent retrospective studies have further clarified the potential association between unilateral/bilateral cortical involvement and epileptogenesis in MOG antibody-associated encephalitis (5). In addition to seizures, patients with MOG-CCE may present with headache, fever, and cerebral cortical symptoms such as aphasia, dysarthria, paralysis, psychiatric symptoms and memory loss, depending on the localization of inflammatory lesions within the CNS (6). Histopathological analyses of cortical tissue specimens demonstrate predominant perivascular lymphocytic infiltration with concomitant microglial activation, suggesting that T-cell-mediated immunopathology predominantly contributes to demyelination mechanisms (7).

The diagnostic confirmation of MOGAD requires detection of MOG-IgG antibodies in both serum and cerebrospinal fluid (CSF) via live cell-based assay, combined with compatible clinical- radiological features and exclusion of alternative diagnoses (8–10). Intriguingly, recent case reports describe the dual positivity of MOG-IgG and anti-N-methyl-D-aspartate receptor (NMDAR) antibodies, emphasizing the diagnostic complexity arising from overlapping autoimmune mechanisms and underscoring the necessity of comprehensive neural antibody profiling to distinguish concurrent autoimmune encephalitides (11). Early initiation of immunotherapy in most patients with MOG-IgG antibodies achieves favorable outcomes. Conversely, delayed treatment may result in symptom progression and poorer prognosis in some cases (12).

These pathophysiological and clinical insights highlight the critical importance of early CSF and serological evaluations, coupled with neuroimaging pattern recognition, to guide timely initiation of targeted immunomodulatory therapies. Our findings corroborate emerging evidence that may constitute a distinct clinical-radiological phenotype, necessitating heightened diagnostic vigilance for early detection.

## Case presentation

A 36-year-old man presented to the emergency department with acute-onset convulsions and mixed aphasia. Five weeks before admission, the patients had experienced a self-limiting febrile illness (peak temperature 39°C) accompanied by diarrhea and myalgia, initially managed as a viral infection. Four weeks later, he developed moderate-intensity stabbing pain localized to the left frontoparietal region without medical evaluation. The patient complained of persistent stabbing pain in the left frontoparietal region. One hour before hospital admission, the patient experienced a generalized tonic-clonic seizure characterized by sudden loss of consciousness with subsequent tonic-clonic movements of all extremities, lasting approximately 5 minutes before spontaneous termination. Postictal global aphasia was observed, manifesting as impaired language comprehension and complete loss of verbal output. Physical examination revealed altered mental status and complete mixed aphasia. Cranial nerves, motor strength, and deep tendon reflexes were normal. An equivocal Babinski sign was noted on the right side, with absence of meningeal signs.

**Figure 1:**
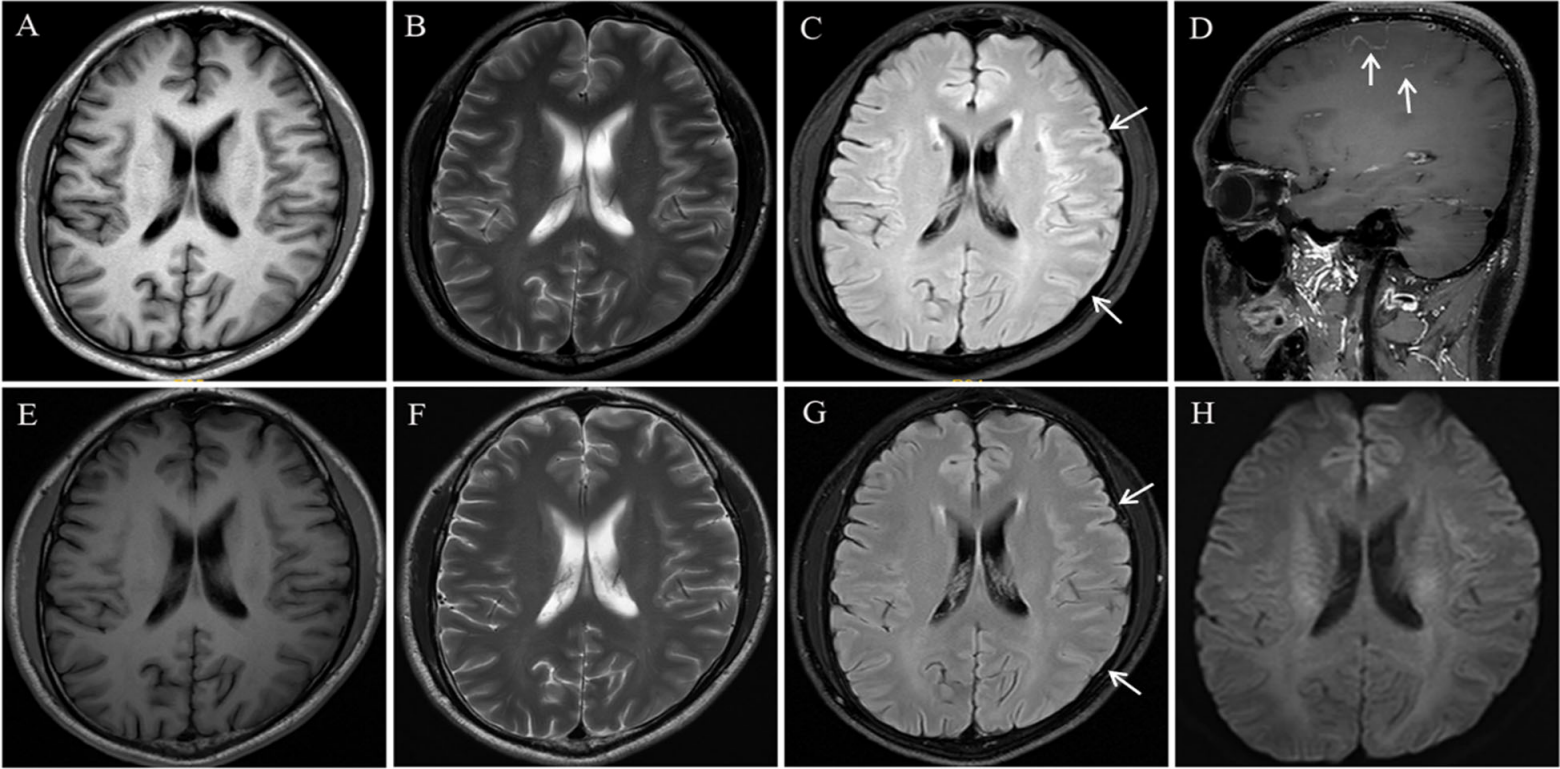
Brain magnetic resonance imaging (MRI): On admission, brain MRI showed no significant signal abnormalities on T1- and T2-weighted sequences **(A, B)**. FLAIR images revealed subtle hyperintensity signal in the right frontoparietal cortex and subcortex, accompanied by gyral swelling and sulcal effacement **(C)**. Post-contrast T1-weighted imaging demonstrated focal leptomeningeal enhancement within the affected region **(D)**. Repeat brain MRI performed at 5-week follow-up demonstrated no abnormal signal on T1- and T2-weighted sequences **(E, F)**, along with significant improvement in FLAIR signal abnormalities accompanied by marked resolution of gyral swelling and sulcal effacement in the frontoparietal regions **(G)**. No abnormal signal was observed on DWI **(H)**.

MRI with contrast revealed no signal alterations on T1-weighted imaging (T1WI ) or T2WI (**Figures 1A, B**), but demonstrated an ill-defined hyperintense signal on Fluid Attenuated Inversion Recovery (FLAIR) sequences in the right frontoparietal lobe, accompanied by gyral swelling and sulcal effacement (**Figure 1C**). Post-contrast T1WI demonstrated focal leptomeningeal enhancement within the affected regions (**Figure 1D**). Video-EEG monitoring, initiated 7 hours post-admission and continued for 24 hours, captured no epileptiform discharges. Laboratory evaluation revealed elevated serum creatine kinase (1169.5 U/L, normal range: 50~310 U/L) and leukocytosis (13.92×10^9^/L, normal range: 4~10×10^9^/L). CSF analysis demonstrated an opening pressure 110 mmH_2_O (normal range: 80~180 mmH_2_O), leukocytosis 24 cells/μL (normal range: 0~8/μL; 95.8% mononuclear), elevated protein level of 70.3 mg/dL (normal range: 8~43mg/dL), and negative oligoclonal bands. Metagenomic sequencing identified 78 Epstein-Barr virus sequence reads. Anti-MOG antibodies were detected in both serum and CSF via cell-based assays (titer 1:32).

The patient was diagnosed with MOGAD presenting as CCE, confirmed by positive anti-MOG antibodies in both serum and CSF. First-line immunotherapy was initiated, including intravenous immunoglobulin (0.4 g/kg/day for 5 days) and methylprednisolone pulse therapy (1g/day for 5 days), followed by an oral prednisolone taper starting at 60 mg/day with weekly reductions of 10 mg. Clinical improvement occurred within 7 days, with complete cessation of seizures and resolution of headaches. Notably, speech improvement observed prior to treatment initiation was attributed to postictal state resolution rather than therapeutic response, as supported by electrical activity suppression of EEG findings.

The patient was discharged after 14 days of hospitalization with no residual neurological deficits on examination. After the 5-week follow-up, serum anti-MOG antibodies remained detectable at a reduced titer of 1:10 using a cell-based assay. Repeat brain MRI revealed no abnormal signal on T1WI and T2WI (**Figures 1E, F**) and significant radiological improvement in cortical swelling and sulcal effacement compared to prior imaging (**Figure 1G**). Diffusion-weighted imaging (DWI) demonstrated no abnormal signal intensity (**Figure 1H**)

## Discussion

MOG-CCE is a rare neuroinflammatory entity within the MOGAD spectrum, presenting unique diagnostic challenges due to its overlapping clinical and imaging features with infectious encephalitis and other autoimmune encephalitis (8). This case highlights the importance of integrating clinical, laboratory, and neuroimaging clues for timely diagnosis.

MOG is a glycoprotein expressed on the surface of myelin sheaths in the central nervous system and functions as a target autoantigen in immune-mediated demyelinating diseases (13). Although the precise mechanisms underlying the generation of anti-MOG antibodies remain incompletely understood, environmental factors such as viral infections are hypothesized to trigger loss of immune tolerance (14). The pathophysiological relationship between EBV infection and MOGAD requires further investigation (15, 16). Molecular mimicry between EBV-derived epitopes and MOG extracellular domains has been reported as a potential mechanism (17), and the temporal correlation between viral illness onset and neurological symptoms in our patient supports infection-induced immune tolerance breakdown. Recent studies report EBV seropositivity rates exceeding 80% in MOGAD cohorts, suggesting its role for EBV in B cell-mediated autoantibody generation (16). Preclinical models further demonstrate that EBV-induced BBB breakdown may allow peripherally produced anti-MOG antibodies to infiltrate the CNS (17). While these findings may explain how peripherally generated antibodies access CNS targets, the mechanisms driving elevated serum MOG-IgG levels in MOGAD remain to be fully elucidated. Notably, rare cases of CSF-restricted MOG-IgG positivity underscore the necessity of comprehensive antibody testing in both serum and CSF using live CBA when clinical suspicion persists despite negative serum results (18). Live CBA represent a reliable method compared to fixed-cell techniques, as they preserve the native conformation of membrane-bound antigens, ensuring accurate epitope recognition and enhanced diagnostic specificity. Anti-MOG antibodies contribute to CNS demyelination through mechanisms that remain incompletely understood. Emerging evidence indicates that CD4+ T cells and B cells act synergistically to amplify demyelination, likely via complement activation and pro-inflammatory cytokine release (7, 19).

The clinical manifestations of MOG-CCE include seizures, headache, fever, focal neurologic deficits, altered mental status, memory impairment, and autonomic dysfunction (6). Among these, seizures (85%) and headache (82%) are frequently reported as predominant features (6), consistent with our patient’s presentation. Although encephalitic forms of MOG-CCE may present with seizures as an initial feature, prodromal headaches occurring weeks prior to overt neurological deficits have recently been recognized as a potential diagnostic marker (6, 20, 21). When accompanied by subtle neuroimaging abnormalities, these prodromal symptoms should raise suspicion for autoimmune CNS disorders such as MOG-CCE.

Neuroimaging, while limited in specificity, offers key clues to differentiate MOG-CCE from clinical mimics. Typical MRI findings include uni-/bilateral cortical/subcortical T2/FLAIR hyperintensities with gyral swelling and leptomeningeal enhancement, these features distinct from classic demyelinating plaques (2, 22). Notably, cortical swelling and sulcal effacement may precede overt signal abnormalities, highlighting the need for thorough evaluation of structural cortical changes even in equivocal scans as seen in our patient. Contrast-enhanced sequences demonstrating meningeal inflammation provide additional diagnostic specificity (23). EEG may demonstrate slow waves or epileptiform discharges. In our case, however, the EEG performed 7 hours after admission showed no evidence of epileptiform discharges. This phenomenon may be attributed to energy depletion from sustained epileptic activity and compensatory upregulation of inhibitory neurotransmitters rather than deep-seated epileptogenic foci, supported by a diffuse low-voltage background with increased slow-wave activity and cortical epileptogenic focus. Repeat EEG following clinical recovery from the postictal state may demonstrate residual epileptiform discharges.

The differential diagnoses required for MOG-CCE include viral encephalitis and autoimmune encephalitis, such as NMDAR encephalitis. In our case, the patient presented with headache and generalized tonic-clonic seizures accompanied by transient postictal fever (38.5°C), initially misdiagnosed as viral encephalitis despite neuroimaging revealing predominant frontoparietal lobar swelling. However, viral encephalitis, particularly herpes simplex virus encephalitis (HSVE), typically manifests with scattered lesions and is pathognomonically characterized by preferential involvement of the medial temporal lobes, insular regions, orbitofrontal cortex, and cingulate gyrus, frequently accompanied by hemorrhagic transformation in severe cases (24). In contrast, MOG-CCE generally presents with confluent cortical lesions and lacks intracranial hemorrhage. Atypical cases warrant the integration of autoantibody testing and next-generation sequencing (NGS) to achieve accurate differential diagnosis. Although over half of anti-NMDAR encephalitis cases may exhibit abnormal signals on T2-weighted or FLAIR sequences, only a minority involve the cortex, typically manifesting as cortical enhancement (25). This pattern contrasts with the leptomeningeal enhancement observed in MOG-CCE (**Table 1**).

**Table 1:** Identification of MOG-CCE with HSVE and NMDAR.

Disease	MOG-CCE	HSVE	NMDAR
Clinical manifestations			
Common clinical features Additional clinical features	Seizures, Headache Fever Mental status changes Altered consciousness Aphasia, Dysarthria Paralysis	Fever, Headache Mental behavior changes Seizures Cognitive dysfunction Altered consciousness Language disorder	Seizures Extrapyramidal symptoms Psychosis Altered consciousness Language abnormality Sleep disturbance
MRI features			
Lesion location Structural abnormalities Signal characteristics Enhancement Pattern **Diagnostic biomarkers**	Unilateral cortex Bilateral cortex Gyral swelling Sulcal effacement T1WI: No obvious changes T2WI: No obvious changes FLAIR: Hyperintense DWI: No obvious changes Leptomeningeal Serum/CSF MOG-IgG	Temporal lobe Insular regions Orbitofrontal cortex Cingulate gyrus Swelling of the affected area T1WI: Hypointense T2WI: Hyperintense FLAIR: Hyperintense DWI: Hyperintense Affected area Serum/CSF NMDAR-IgG	Medial temporal lobes Cerebral cortex Cerebellum Brainstem, Basal ganglia Absence or swelling of the affected area No signal abnormalities or Hypointense on T1WI and Hyperintense on T2WI, FLAIR, and DWI Absence or the affected area Virus nucleic acid sequence

MOG-CCE, Myelin oligodendrocyte glycoprotein-associated cerebral cortical encephalitis; HSVE, herpes simplex virus encephalitis; NMDAR, anti-N-methyl-D-aspartate receptor; T1WI, T1-weighted imaging; T2WI, T2- weighted imaging; FLAIR, Fluid Attenuated Inversion Recovery; DWI, Diffusion-weighted imaging.

MOG-CCE generally has a more favorable prognosis with early intervention. First-line treatment for the disease is intravenous methylprednisolone, followed by an oral taper (3). For patients with severe clinical condition or inadequate response to corticosteroid therapy, intravenous immunoglobulin or therapeutic plasma exchange should be considered (26). Timely immunosuppressive therapy significantly reduces inflammatory demyelination, the occurrence of serious complications, and improves recovery in the majority of MOG-CCE patients. However, relapse prevention remains challenging. A retrospective analysis of 124 patients with a definite MOGAD diagnosis revealed that 50 (40.3%) cases presented with a monophasic disease course, whereas 74 (59.7%) experienced relapses. The median time interval from disease onset to the first relapse was 3 months, suggesting that immunotherapy should be continued for at least 3 months or longer, depending on the specific clinical situations (27).

Risk factors for disease relapse in MOGAD include older age at onset, initial presentation with transverse myelitis or encephalitis, severe initial attacks, incomplete recovery, frequent relapses, and high titers of MOG antibodies (12, 28, 29). Emerging evidence suggests that TNF-alpha-induced protein 3 (TNFAIP3), a regulator of inflammatory signaling pathways, may serve as a potential biomarker for relapse. Clinical studies revealed significantly reduced TNFAIP3 levels during relapses compared to remission phases in patients with MOGAD (30); however, its clinical utility remains investigational. A previous study demonstrated that patients receiving immunosuppressive therapy for ≥3 months after disease onset had a significantly lower relapse risk than those with shorter or no immunosuppression. This underscores the critical role of maintenance therapy in relapse prevention (31). Current guidelines support prolonged immunosuppression with agents such as mycophenolate mofetil or rituximab, particularly in patients with high-titer MOG antibodies or polysymptomatic presentations. These findings emphasize the importance of biomarker-guided therapeutic strategies, including monitoring dynamic biomarkers like TNFAIP3 expression.

## Conclusion

This case highlights that MOG-CCE should be considered in patients presenting with seizures and persistent headache when subtle structural MRI changes such as gyral swelling or sulcal effacement are observed, even without overt signal abnormalities. Early detection of these clinical and imaging features may provides critical diagnostic clues for MOG-CCE and justify prompt MOG-IgG antibody testing in both serum and CSF to establish a definitive diagnosis. MOG-IgG antibody testing is also critical to distinguish MOG-CCE from viral or autoimmune encephalitis, given the frequent misdiagnosis in clinical practice. Immediate immunotherapy induces complete remission in most patients, those with relapsing disease require long-term immunotherapy. Future research should focus on standardization of diagnostic criteria and validation of relapse-predictive biomarkers to facilitate timely diagnosis, targeted therapy, and prevention of recurrent attacks, ultimately reducing neurological disability in MOG-CCE.

## Data Availability

The raw data supporting the conclusions of this article will be made available by the authors, without undue reservation.
